# Bridging the gap: unleashing the power of non-core departments through interdepartmental collaboration

**DOI:** 10.3389/fpsyg.2023.1275666

**Published:** 2024-01-15

**Authors:** Siyu Ren, Yile Wang

**Affiliations:** ^1^Financial Department, Sichuan University, Chengdu, Sichuan, China; ^2^Business School, Sichuan University, Chengdu, Sichuan, China

**Keywords:** department diversity, non-core department, knowledge spillover, firm performance, China

## Abstract

Traditional research on firm performance has predominantly emphasized the role of key departments, often underestimating the potential contributions of non-core departments. This study redresses this oversight by investigating the impact of non-core departments on firm performance. Utilizing a comprehensive 20-year dataset from Chinese A-listed firms and employing the endogenous growth model, we scrutinize the influence of non-core departments on enterprise productivity and organizational growth. Our findings underscore that non-core departments significantly enhance firm performance. Furthermore, we observe a negative coefficient of the interaction term, implying the presence of diminishing returns to scale when amalgamating department diversity with firm knowledge. This suggests that while both department diversity and firm knowledge independently contribute positively to firm performance, their conjoined effect does not necessarily induce a proportionally amplified impact. Moreover, we found that factors such as the company’s equity structure, market environment, and the age and education level of executives may moderate the impact of departmental diversity on firm performance. This study enriches the literature by spotlighting the potential of non-core departments in propelling firm success and underlines the imperative for strategies that cultivate interdepartmental collaboration. The implications of these findings propose that firms can leverage the potential of non-core departments for sustainable growth, offering a fresh perspective for future research in organizational development.

## 1 Introduction

In the complex business landscape, non-core departments such as customer service, public relations, and human resources are often undervalued. These departments, traditionally seen as supportive, play a crucial role in shaping firm performance. Their unique roles, skills, and experiences contribute significantly to an organization’s resilience and adaptability. They stimulate innovation and enhance efficiency by fostering interdepartmental collaboration. When integrated with core units, they amplify functionality, propelling the organization forward.

In periods of economic contraction, firms frequently streamline operations by downsizing or eliminating peripheral departments to concentrate on core functions. Yet, empirical evidence suggests that such departments can be instrumental in enhancing organizational performance. For example, BYD, a frontrunner in the automotive and new energy sectors, leverages its logistics department to achieve cost-efficiency and competitive advantage through automation and supply chain optimization. This case underscores the potential for non-core departments to contribute significantly to corporate resilience, even in adverse economic conditions.

However, the potential of non-core departments in enhancing organizational diversity and firm performance has not been fully realized. This is partly due to a lack of understanding and appreciation of their role and potential. The existing literature presents a multifaceted relationship between departmental structure and organizational performance, offering both opportunities and challenges (Homan et al., 2015; Zahoor et al., 2023). The unique skills, experiences, and perpectives within departments can serve as catalysts for creativity and problem-solving, potentially enhancing firm performance. This effect is particularly pronounced when collaboration between non-core and key departments is fostered, as such collaboration engenders a diversity of thought and approach that can be highly beneficial for firm decision-making. However, interdepartmental collaboration, while a strength, can also introduce complexities. Differences in functions and responsibilities across departments can lead to conflicts and communication barriers, potentially negatively impacting team cohesion and productivity (Wissen Hayek et al., 2016).

To delve deeper into the role and impact of non-core departments on firm performance, we utilize a comprehensive 20-year dataset collected from numerous listed firms in China. Our research applies endogenous growth models to delve into non-core departments’ internal mechanisms and effects. Through empirical testing of the proposed model, we find that departmental diversity significantly boosts firm performance. This positive association persists even after controlling for reverse causality and employing the number of local universities as an instrumental variable to address potential endogeneity issues. Interestingly, we also observe a negative coefficient for the interaction term between departmental diversity and firm knowledge, indicative of diminishing returns to scale when these two facets are amalgamated.

These findings provide compelling evidence underscoring the pivotal role of non-core departments in enhancing firm productivity and performance. They also highlight the necessity of considering firm-specific and environmental factors when leveraging the benefits of departmental diversity. This nuanced understanding can guide firms in strategically utilizing their non-core departments to achieve sustainable growth and competitiveness.

In conclusion, our research expands the existing literature on organizational structure by providing a comprehensive understanding of the impact of non-core departments on organizational resilience. By focusing on the internal mechanisms that drive the benefits of non-core departments, we underscore the importance of viewing these departments as strategic assets in a competitive market landscape. Unlike executive diversity, which primarily influences strategic decision-making and organizational direction, structural diversity encompasses various levels and departments within a firm, thereby exerting a more pervasive impact on operational and tactical decisions. We aim to provide valuable insights that can help organizational leaders, human resource managers, and policymakers in their efforts to optimize organizational structure and performance.

## 2 Literature review

Non-core departments, traditionally seen as secondary, are now acknowledged as key resources impacting operations and strategic capabilities. These departments, such as Human Resources, Information Technology, Legal, and others, offer specialized knowledge and skills crucial to a firm’s operation (Messersmith et al., 2011; Chen et al., 2022).

These departments cultivate functional diversity, enhancing problem-solving, decision-making, and operational precision. By leveraging their unique competencies, organizations can optimize processes, remove redundancies, and realize cost savings. Individuals in these departments, due to their distinct roles and experiences, foster cognitive diversity. This diversity, marked by variations in problem perception and approach, can augment the firm’s knowledge base, bolstering its innovation and problem-solving ability (Aggarwal et al., 2019).

Moreover, non-core departments enhance intra-organizational communication and collaboration, encouraging interdepartmental learning and cross-functional cooperation. This interaction enables knowledge spillover, utilizing the advantages of functional and cognitive diversity to propel the enterprise’s endogenous technological advancement, strengthening its competitive position.

Before delving into the analysis, it’s crucial to examine existing research on diversity. This ensures a thorough understanding of the current discourse, allowing us to situate our study within this context and pinpoint areas for unique insights.

### 2.1 The multiple aspects of diversity in current research

The existing body of literature emphasizes the critical role of diversity in management echelons and boards in shaping firm performance dynamics. It illustrates how diverse management teams and boards promote organizational innovation and improve financial performance by generating a multitude of ideas and solutions, thereby enriching the strategic decision-making process (Certo et al., 2006; Campbell and Minguez-Vera, 2008; Miller and Triana, 2009). This leads to the recognition that managerial diversity can affect firm performance indirectly. Empirical evidence also shows that a diverse management team can improve a firm’s standing among various stakeholder groups and thus improve performance (Brammer et al., 2007).

The focus of research has been on diversity attributes such as race, gender, nationality, and educational background. For instance, Hoogendoorn et al. (2013) conducted a field experiment to assess the impact of gender diversity on business teams on their performance and found that teams with an equal gender mix performed better than male-dominated teams in terms of sales and profits.

Some studies have also delved into the complex relationship between cultural diversity and firm performance, suggesting that this relationship can be mediated by aspects of entrepreneurial orientation, including innovativeness, risk-taking, and proactivity (Richard, 2004). Khatib et al. (2021) conducted a systematic review of board diversity in financial institutions, highlighting the need to explore other board diversity attributes besides gender. Sheehan and Anderson (2015) call for research on talent management and organizational diversity, emphasizing the impact of diversity on sustainable development.

A conspicuous lacuna in extant literature is the insufficient focus on departmental diversity, which serves as the foundational layer of organizational diversity and exerts a broad impact at lower hierarchical levels. Investigating diversity at the departmental level offers a novel lens through which to understand the intricate relationship between diversity and organizational performance. This underexamined dimension is crucial for a comprehensive understanding of how workforce diversity influences firm outcomes. Hence, it is within this framework that the imperative for a more expansive examination of workforce diversity gains prominence.

### 2.2 The geographical focus in current diversity research

A review of the existing body of literature investigating the interconnections between diversity and firm performance discloses a marked geographic bias towards Europe and other Western nations (Ali et al., 2011). Southern Europe, too, has been a focal point of scholarly attention, with research assessing the impact of board diversity on firm value in Spain (Campbell and Minguez-Vera, 2008). Even when considering the global landscape, the research focus remains predominantly Western, as exemplified by studies on diversity-performance dynamics within European-based multinational corporations (MNCs) (Randel et al., 2018). These studies underscore the essential role of diversity within globally dispersed teams and its consequential influence on MNC performance.

This Western and European-focused lens in existing research amplifies the need for exploratory investigations in other geographical contexts. In particular, understanding the diversity-performance relationship in Asian economies, such as China, becomes increasingly important. China provides a unique context due to its rapid economic growth, diverse industrial sectors, and the government’s emphasis on innovation and technology. Such research expansions would contribute to a more inclusive, globally representative, and comprehensive understanding of the department diversity and firm performance relationship.

Our subsequent analysis will delve into the mechanisms through which the diversity fostered by non-core departments influences a firm’s performance. In line with the findings of Albitar et al. (2020), we posit that departmental diversity, as an internal factor within a firm, can significantly contribute to its growth. This is further explored through the lens of the endogenous growth model, a theoretical framework that enables us to examine such internal factors.

In line with the Resource-Based View (RBV) theory, we argue that departmental diversity is a unique and invaluable asset with direct implications for an organization’s revenue streams. RBV posits that resources that are rare, valuable, and non-substitutable confer a sustainable competitive advantage. Within this framework, knowledge spillovers generated by departmental diversity serve a dual purpose. First, they enhance problem-solving capabilities by introducing a variety of perspectives and approaches, thereby enriching the organization’s intellectual capital. Second, these spillovers act as a catalyst for cross-departmental collaboration, leading to the development of new products, services, or processes that could be proprietary to the firm. This not only amplifies the firm’s resource base but also creates avenues for sustainable competitive advantages and new revenue streams. Empirical evidence from Breschi and Lissoni (2009) and Trax et al. (2015) further substantiates the revenue-enhancing potential of diversity, highlighting its role in magnifying knowledge spillovers and boosting total factor productivity.

To guide our forthcoming econometric analysis, we formulate hypothesis 1(H1), which posits that departmental diversity exerts a positive influence on firm performance. This hypothesis is grounded in the premise that a diverse department, through interactive assistance, can effectively invigorate core departments. By bringing a wealth of perspectives, ideas, and strategies to the table, these diverse departments can foster innovation and improve decision-making processes. This collaborative interaction not only enhances the functionality of core departments but also ultimately boosts the overall performance of the firm. This is supported by the work of Christiansen et al. (2016), who found a positive association between diversity in senior positions and firm performance.

*H1*: Departmental diversity has a positive effect on firm performance.

Furthermore, the knowledge spillover effect, as discussed by Hájek and Stejskal (2018), suggests that the diverse knowledge and experiences within a department can lead to innovation and sustainable performance, thereby positively impacting firm performance. Thus, the potential impact of departmental diversity on firm performance is multifaceted and warrants further investigation.

### 2.3 The multifaceted impact of departmental diversity on organizational performance

The convoluted nature of departmental diversity within an organization offers substantial opportunities but also brings potential hurdles when intertwined with a firm’s existing knowledge base (Dufays and Huybrechts, 2016; Guillaume et al., 2017; Martinez et al., 2017). The rich tapestry of diversity within departments can cultivate an environment conducive to learning. The interplay of diverse experiences, insights, and perspectives promotes an atmosphere of reciprocal support, fostering a climate of inclusivity and collaboration. Brewer (1991) have pointed out the importance of this balance in organizations.

This harmony of diversity allows for the simultaneous nurturing of a collective identity while enabling individual uniqueness. Employees can affiliate themselves with the larger team while maintaining their distinct identities since the sense of belonging and value is a key driver of productivity and enhances overall performance (Nishii, 2013), an arrangement that furthers departmental integration and underpins a cooperative and inclusive ambience (Shore et al., 2011). The ramifications of this environment reach far beyond individual departments. The constant exchange of new ideas and information, known as the spillover effect, complements the organization’s existing knowledge reserves. This blend of old and new can significantly enhance the firm’s operational efficiency and overall competency, effectively harnessing the power of diversity to fuel performance (Herring, 2009).

Nevertheless, the potential benefits of diversity remain under the condition that diversity is skillfully managed, and organizations consciously nurture an inclusive culture (Guillaume et al., 2017). Without this crucial aspect, diversity risks becoming a factor of discord within the company, clashing with its established knowledge base. This conflict often manifests as misunderstandings and miscommunications, stemming from diverse communication styles and cultural expressions within the workforce (Cox and Blake, 1991). Such discrepancies, if left unaddressed, can spark intra-team conflicts and disrupt cooperative dynamics, eventually compromising organizational performance.

The situation becomes more complicated when departmental diversity and firm knowledge are not effectively coordinated (Chatman and O'Reilly, 2004). The consequence is often the emergence of information and knowledge silos, which segregate rather than integrate, leading to the fragmentation of the firm’s collective knowledge base. This complication not only impedes the organization’s fluid operation but may also lead to potentially detrimental effects, signaling the possible onset of diminishing returns to scale (van Knippenberg and Mell, 2016). The interaction effect of department diversity and the stock of knowledge becomes less beneficial, influenced by a variety of factors such as management practices, the industrial environment, and the broader sociocultural context.

To better understand these intricate dynamics, we propose two hypotheses. H2a posits that the interplay between departmental diversity and firm knowledge has a positive effect on organizational performance. Conversely, H2b suggests that this interaction may lead to a significant decline in firm performance. By evaluating these hypotheses through rigorous econometric analysis, we seek to provide a more empirical understanding of the critical role the interaction between departmental diversity and firm knowledge plays in shaping firm performance. This research will have relevance for Chinese A-share listed firms, offering them insights into managing diversity effectively.

*H2a*: The interaction between departmental diversity and firm knowledge has a positive effect on firm performance.

*H2b*: The interaction between departmental diversity and firm knowledge has a detrimental effect on firm performance.

## 3 Methodology

### 3.1 Model

The extant corpus of literature investigating the correlation between diversity and firm performance presents a variety of methodological methodologies, predominantly oriented towards empirical techniques. These strategies are pivotal for deciphering the complex dynamics between diversity and its consequent influence on firm performance. Despite the myriad of methodologies employed in previous studies, certain theoretical lenses remain conspicuously underexplored. The learning-by-doing model proposed by Arrow (1962) suggests that an increase in experience and knowledge acquisition yields augmented productivity at both individual and firm levels. This presents a fresh perspective on the diversity-performance relationship within firms. Our research concludes that fostering interdepartmental collaboration, especially among non-core departments, can instigate knowledge dissemination, thereby bolstering organizational output. As these employees persistently derive insights from their undertakings, the overall ability reaps the benefits of this knowledge accretion, translating into heightened output and sustained growth. Thus, we posit that the learning-by-doing model can proffer additional insights into the dynamics of organizational performance.

The most common way to address the impact of department diversity on production is to start with the Cobb–Douglas function:

Equation 1: Cobb–Douglas Production Function.


(1) $$Y=A*K^{\alpha }*L^{\left(1-\alpha \right)}$$

Where:

Y = total output.

A = the level of technology (total factor productivity).

K = the stock of physical capital.

L = labor input.

α = the share of output attributable to capital (0 < α < 1).

In equation 1, the level of the level of technology, represented as total factor productivity (A), is determined endogenously within the economy. This equation recognizes that knowledge spillovers can significantly influence total factor productivity.

In this study, we extend this understanding by incorporating the knowledge spillover effect into the Cobb–Douglas production function. We denote the knowledge spillover effect as (S) and conceptualize it as an interaction between a firm’s knowledge output (H) and departmental diversity (D). This equation is premised on the idea that departmental diversity can enhance the dissemination and application of a firm’s knowledge output, thereby catalyzing knowledge spillovers. We define the knowledge spillover effect (S) as the interaction between a firm’s knowledge output (H) and departmental diversity (D), represented as:


$$S={\left(H*D\right)}^{\beta_{3}}$$

This conceptualization is informed by Raimbault (2022) work, which highlights the role of informal knowledge exchanges and interactions between diverse firms in fostering innovation. Raimbault’s findings suggest that the interaction between diverse entities and knowledge output can lead to a synergistic effect, enhancing the overall innovation process.

In line with this, we posit that a similar interaction between H and D in our context can generate a synergistic effect, where the combined influence on knowledge spillovers surpasses the sum of their individual effects. This interaction, represented by the multiplicative term (H*D), captures the idea that the impact of departmental diversity on knowledge spillovers is not merely additive but is amplified when combined with the firm’s knowledge output. The parameter β3 in the equation then indicates the extent of this amplification effect on the spillover effect.

To incorporate this knowledge spillover effect into the equation for total factor productivity (A), we propose the following formulation:


$$A=H^{\beta_{1}}*D^{\beta_{2}}*{\left(H*D\right)}^{\beta_{3}}$$

Where:

A = total factor productivity.

H = stock of knowledge.

D = department diversity.

In this equation, the parameters $\beta_{1}$ , $\beta_{2}$ , and $\beta_{3}$ capture the effects of H, D, and their interaction on A, respectively. This formulation implies that total factor productivity (A) is a function of the firm’s knowledge output (H), department diversity (D), and the interaction between H and D.

Our model is corroborated by empirical research. For instance, studies by Parrotta et al. (2014), Nathan and Lee (2013) have demonstrated a positive association between these variables. These studies collectively suggest that departmental diversity within a firm can catalyze knowledge spillovers, thereby enhancing total factor productivity. Therefore, our model offers a theoretical framework for comprehending the intricate interplay between departmental diversity, knowledge spillovers, and firm productivity. It posits that the knowledge spillover effect, represented by the interaction between a firm’s knowledge output and departmental diversity, plays a crucial role in enhancing total factor productivity. This theoretical framework provides a nuanced understanding of the mechanisms through which departmental diversity can contribute to firm productivity, thereby laying a foundation for further empirical investigation.

Finally, we incorporate our newly defined expression for total factor productivity (A) into the Cobb–Douglas production function, resulting in the following equation 2:

Equation 2:


(2) $$Y=H^{\beta_{1}}*D^{\beta_{2}}*{\left(H*D\right)}^{\beta_{3}}*K^{\alpha }*L^{\left(1-\alpha \right)}$$

To facilitate the application of linear regression methods for model estimation, we linearize the equation 2 by taking the natural logarithm of both sides:


$$\begin{array}{c} \ln\left(Y\right)=\beta_{1}*\ln\left(H\right)+\beta_{2}*\ln\left(D\right)+\beta_{3}*\ln\left(H\right)*\ln\left(D\right)\\ +\;\alpha *\ln\left(K\right)+\left(1-\alpha \right)*\ln\left(L\right) \end{array}$$

This transformed equation enables us to estimate the individual effects of firm knowledge output (H) and department diversity (D) on total output (Y). Additionally, it allows us to quantify their interaction effect, which encapsulates the impact of department diversity on knowledge spillover.

Rearrange we have Equation 3:


(3) $$\begin{array}{c} \ln\left(Y\right)=\beta_{0}+\beta_{1}*\ln\left(H\right)+\beta_{2}*\ln\left(D\right)+\beta_{3}*\ln\left(H\right)*\ln\left(D\right)\\ +\;\alpha *\ln\left(K\right)+\left(1-\alpha \right)*\ln\left(L\right)+\varepsilon \end{array}$$

$\beta_{1}$ : The coefficient for ln(H) captures the direct effect of firm knowledge on firm revenue. If $\beta_{1}$ is positive and statistically significant, it suggests that an increase in firm knowledge is associated with an increase in firm revenue, holding all other factors constant.

$\beta_{2}$ : The coefficient for ln(D) represents the direct effect of departmental diversity on firm revenue. A positive and statistically significant $\beta_{2}$ implies that an increase in departmental diversity is associated with an increase in firm revenue, holding all other factors constant. This suggests that departmental diversity can contribute to firm revenue independently of its interaction with firm knowledge.

$\beta_{3}$ : The coefficient for ln(H)*ln(D) represents the interaction effect between firm knowledge and departmental diversity on firm revenue. This interaction effect captures the knowledge spillover effect, which is the additional impact on firm revenue resulting from the synergistic interaction between firm knowledge and departmental diversity. A positive and statistically significant $\beta_{3}$ indicates that the knowledge spillover effect enhances firm revenue. In other words, the combined effect of firm knowledge and departmental diversity on firm revenue is greater than the sum of their individual effects.

### 3.2 Sample

Our analysis concentrates on the performance of China’s A-share listed firms, chosen due to the vast geographic diversity and the variety of regional dialects, customs, and norms this country encompasses. The period from 2001 to 2020 is significant as it marked China’s entry into the World Trade Organization (WTO) in 2001, instigating a profound economic transformation. Post this event, the country saw an impressive increase in both the scale of its economy and the size and number of listed firms. This evolving economic environment underlines the importance of investigating the influence of department diversity on firm performance.

Considering the inherent heterogeneity characterizing the core operations across diverse industries—including but not limited to aviation, tourism, and various service sectors—our empirical investigation strategically narrows its focus to ‘production enterprises’. These are firms that explicitly disclose the presence of production departments and largely align with the organizational structure of traditional manufacturing entities. Notably, such firms constitute approximately 88% of our total sample. This methodological choice serves a dual purpose: it not only mitigates selection bias, thereby bolstering the external validity of our findings, but also accommodates inter-industry variability by concentrating on a subset of firms with similar organizational architectures.

To ensure the robustness and validity of our sample selection, we employed a multi-pronged approach: (1) Exclusion of firms from the financial sector to eliminate industry-specific volatility and regulatory impacts. (2) Omission of firms with a debt-to-asset ratio exceeding 1 to ensure financial stability and solvency within the sample. (3) Disqualification of firms with significant data deficiencies to maintain the integrity and reliability of the dataset.

Data for this study were meticulously sourced from the WIND and CSMAR databases, adhering to stringent data quality standards. As a result, our final sample comprises 3,365 firms listed on China’s A-share market, yielding a total of 32,136 firm-year observations spanning the years 2001–2020.

### 3.3 Variables

In our model, the dependent variable Y, representing a firm’s income, is introduced. Income is a commonly recognized measure of firm performance as it directly denotes the financial results of a firm’s operational and strategic choices (Novitasari and Agustia, 2021). Additionally, it reflects both internal (such as departmental diversity and knowledge output) and external (like market conditions and competition) influences, offering a holistic view of firm performance.

Diversity, often identified by factors such as educational pedigree, age, gender, and ethnicity, has been quantified in previous literature through numerous indices including, but not limited to, the Fractionalization index (Alesina et al., 2003) and the Shannon–Weaver entropy index (Niebuhr, 2010; Østergaard et al., 2011). When focusing on the unique aspect of departmental diversity within firms, the challenge lies in the limitations of available direct measurement methods. The presence of scoring scales based on questionnaires is acknowledged; however, their inherent subjectivity calls into question their suitability for large-sample studies. Addressing this concern, our investigation seeks to operationalize the measure of departmental diversity by determining the ratio of employees in non-core departments to the total employee count within the firm.

We systematically identify ‘core departments’ as encompassing Sales, Production, Finance, and Technology, a categorization that is undergirded by dual lines of robust rationale: Firstly, our study focuses on production firms listed on China’s A-share market, which are mandated to adhere to stringent regulatory frameworks. Specifically, Article 42 of the ‘Guidelines for the Content and Format of Information Disclosure by Companies Publicly Issuing Securities No. 57—Prospectus’^1^ stipulates that listed companies must disclose employee numbers and professional structures. Articles 43, 45, and 48, along with section 6, encompass areas that are relevant to Sales, Production, Finance and Technology departments, thereby implying an expectation of disclosure or transparency in these sectors. This regulatory uniformity not only ensures internal consistency within our dataset but also enhances cross-sectional comparability, thereby bolstering the empirical rigor of our investigation. Moreover, this compliance with legal stipulations lends credence to our definitional framework, aligning it with both industry norms and regulatory expectations.

Secondly, these four departments are often the linchpins of a firm’s operational and strategic capabilities. They represent the core business functions directly involved in value creation, risk management, and exerting a direct influence on both profitability and market positioning. Particularly after excluding non-production firms, the relatively uniform departmental structure among production firms serves as a primary indicator for external stakeholders to assess the overall health and strategic direction of the company.

Consequently, any department not encompassed within these four categories is, by default, classified as a ‘non-core department’ in our study. These non-core departments, therefore, are posited as instrumental in providing an indicative measure of a firm’s intensity of departmental diversity.

Several control variables are considered to account for other possible heterogeneity factors impacting firm performance: Capital Structure *(Lev)*, the debt-to-asset ratio; Regulatory Capacity *(Ind)*, the proportion of independent directors; Profitability *(Roe)*, the net profit to average net assets ratio; Future Investment Opportunities *(Tbq)*, the ratio of a firm’s market value to its net assets at year-end; Degree of Duty Performance *(Meet),* annual board meetings number; Equity Concentration *(H5),* the total shareholding ratios of the top five shareholders. Furthermore, since *lnK* and *lnI* are not primary explanatory variables, they are incorporated as control variables. All variables are detailed in Table 1. We implemented stringent data-cleaning procedures to ensure result accuracy and reliability. Acknowledging outliers’ potential effect on our regression results, we applied winsorization at the 1st and 99th percentiles to all continuous variables, thereby containing extreme values without excluding them. This mitigates outlier influence while maintaining data integrity. This practice is widely accepted in statistical analysis.

**Table 1:** Variable definition and description.

Variable type	Variable symbol	Variable name	Meaning description
Interpreted variable	*lnY*	Firm income	Total operating income, take natural logarithm.
Explanatory variables	*lnH*	Firm knowledge	R & D investment, take natural logarithm.
	*lnD*	Department diversity	Number of non-core department employees/Total number of employees, take natural logarithm.
Control variables	*lnK*	Capital	Fixed asset, take natural logarithm.
	*lnL*	Number of labors	The number of labors in the firm, takes natural logarithm.
	*Lev*	Capital structure	The ratio of a firm’s total liabilities to its total assets.
	*Ind*	Regulatory capacity	The proportion of independent directors within a firm.
	*Roe*	Profitability	The ratio of net profit to average net assets.
	*Tbq*	Future investment opportunities	The ratio of a firm’s market value to its net assets at year-end.
	*Meet*	Degree of duty performance	The annual number of board meetings held by the firm.
	*H5*	Equity concentration	The sum of the shareholding ratios of the firm’s top five shareholders.

In Table 2, the descriptive statistics for all variables are depicted. Out of a total of 32,136 observations, the average value for departmental diversity, denoted by *lnD,* is established at 0.123, reaching a maximum value of 0.564. This considerable variation reflects a significant level of heterogeneity within the sample, demonstrating a wide array of departmental diversity degrees amongst the firms incorporated in the study. On the other hand, the average value indicates a generally moderate level of departmental diversity across the sampled firms. This diversity and disparity provide an extensive backdrop for analyzing the implications of departmental diversity on firm performance.

**Table 2:** Summary statistic.

Variable	*N*	Mean	Min	Median	Max	Std. dev.
*lnY*	32,136	12.087	0	11.959	15.806	1.426
*lnH*	32,136	5.917	0	7.768	11.486	4.088
*lnD*	32,136	0.123	0	0.107	0.564	0.105
*lnK*	32,136	10.960	0	10.887	14.762	1.632
*lnL*	32,136	7.621	2.079	7.578	10.591	1.234
*Lev*	32,136	0.428	0.052	0.426	0.889	0.203
*Ind*	32,136	0.369	0	0.333	0.571	0.054
*Roe*	32,136	0.071	−0.636	0.076	0.390	0.126
*Tbq*	32,136	1.912	0.880	1.529	7.961	1.164
*Meet*	32,136	9.287	2	9	58	3.885
*H5*	32,136	54.452	20.159	55.022	88.718	15.133

In this empirical study, we prudently apply ‘year’ and ‘industry’ controls, crucial in performance-based analyses due to their notable impact on firm performance. The ‘year’ control accommodates temporal dynamics such as societal shifts, economic variations, policy alterations, and technological advancements, while the ‘industry’ control adjusts for industry-specific characteristics like competitiveness, growth patterns, and labor practices. By ensuring the relationship between department diversity and firm performance is attributed to these elements rather than other factors, we bolster the validity and precision of our findings.

Finally, equation 4 used in the empirical analysis is derived from equation 3 as follows:

Equation 4:


(4) $$\begin{array}{c} \ln Y_{i,t+1}=\beta_{0}+\beta_{1}*\ln H_{i,t}+\beta_{2}*\ln D_{i,t}+\beta_{3}*\ln H*\ln D_{i,t}\\ +\;\sum \beta_{j}*\mathrm{Contro}l_{i,t}+\sum \mathrm{Year}+\sum \mathrm{Industry}+\varepsilon_{i,t} \end{array}$$

To manage potential endogeneity issues resulting from reverse causality, where firm performance could influence departmental diversity, we shift the dependent variable forward by one period. This lagged model simultaneously mitigates reverse causality bias and recognizes that departmental diversity’s effects might manifest over time, rather than instantaneously. This method boosts the robustness of our results, strengthening the validity of our analysis’s causal inferences.

Additionally, the error term $\varepsilon_{i,t}$ might exhibit heteroscedasticity and autocorrelation, potentially underestimating the true standard deviation with traditional estimators, leading to excessive null hypothesis rejection. To mitigate this, we use robust standard errors with the firm as the clustering unit, ensuring the reliability of our statistical inferences.

## 4 Empirical results

### 4.1 Main results

The empirical results, as per the established model, are detailed in Table 3. Column (1) displays the results incorporating only the explanatory variables and fundamental production factors. Column (2) extends the model by including control variables, and Column (3) further refines the model by controlling for year and industry-fixed effects. The coefficients for *lnD_i,t_* across these models are 1.868, 1.499, and 1.145, respectively, and are all statistically significant at the 1% level. These results provide robust evidence of a positive relationship between departmental diversity and firm performance, thereby substantiating Hypothesis 1b. This robust evidence underscores the critical role that diversity within non-core departments plays in enhancing firm productivity. By fostering a diverse workforce, firms can enhance their productivity, thereby achieving sustainable growth and competitiveness in the market.

**Table 3:** Main result: departmental diversity and firm performance.

	(1)	(2)	(3)
Variables	*lnY_i,t + 1_*	*lnY_i,t + 1_*	*lnY_i,t + 1_*
*lnH_i,t_*	0.046***	0.064***	0.054***
	(0.004)	(0.003)	(0.005)
*lnD_i,t_*	1.868***	1.499***	1.145***
	(0.169)	(0.137)	(0.173)
lnH_i,t_*lnD_i,t_	−0.151***	−0.145***	−0.094***
	(0.024)	(0.020)	(0.023)
*lnK_i,t_*	0.301***	0.256***	0.253***
	(0.015)	(0.014)	(0.016)
*lnI_i,t_*	0.586***	0.478***	0.485***
	(0.020)	(0.018)	(0.019)
*Lev_i,t_*		1.529***	1.347***
		(0.074)	(0.080)
*Ind_i,t_*		0.261	0.027
		(0.166)	(0.159)
*Roe_i,t_*		2.107***	2.054***
		(0.077)	(0.076)
*Tbq_i,t_*		−0.072***	−0.075***
		(0.009)	(0.010)
*Meet_i,t_*		0.021***	0.015***
		(0.003)	(0.002)
*H5_i,t_*		0.005***	0.004***
		(0.001)	(0.001)
Constant	4.056***	4.103***	4.174***
	(0.110)	(0.124)	(0.186)
Year	NO	NO	YES
Industry	NO	NO	YES
Observations	32,136	32,136	32,136
*R*-squared	0.645	0.713	0.743

Robust standard errors in parentheses ***p < 0.01, **p < 0.05, *p < 0.1.

In diverse teams, the amalgamation of a wide array of skills, ideas, and perspectives creates a fertile ground for innovation and learning opportunities. This diversity, particularly within non-core departments, fosters an environment of cooperation and mutual learning. As team members interact and collaborate, they learn from each other’s unique skills and perspectives, leading to a collective enhancement of knowledge and competencies. This process of continuous learning and improvement, often referred to as the learning-by-doing effect, results in the refinement of ideas and knowledge over time. The surge in productivity, driven by this cooperative learning environment and the innovative solutions it engenders, manifests in heightened output. This increased output, in turn, culminates in a marked rise in income for the firm.

The empirical data reveals a negative coefficient for the interaction term (lnH_i,t_*lnD_i,t_), indicating a complex relationship between departmental diversity and firm knowledge. While both departmental diversities, with its varied experiences and perspectives, and firm knowledge, which enables informed decision-making, are individually beneficial to a firm’s performance, their combined impact does not proportionally enhance output. This could be the performance-enhancing effect of departmental diversity is less pronounced in firms with high levels of research and development (R&D) investment compared to those with lower levels. In other words, the knowledge spillover effects of departmental diversity might be more beneficial for firms that are not traditionally innovation intensive. For firms that already have substantial R&D investment, the marginal performance improvement brought about by diversity may not be as significant. This finding provides empirical support for Hypothesis 2b, which posits that the complex interplay between departmental diversity and firm knowledge can have a significant negative impact on firm performance.

### 4.2 Robustness checks

Due to the inability to eliminate reverse causality through the interlacing of variables (Bellemare et al., 2017), we adopt a cross-lagged dynamic panel approach, as suggested by Maghyereh and Abdoh (2020), to address this issue. Specifically, we introduce the lagged dependent variable as an explanatory variable and employ the System Generalized Method of Moments (SYS-GMM) for estimation, as proposed by Arellano and Bover (1995) and Blundell and Bond (1998). This method initially utilizes differences to eliminate individual-specific effects and subsequently employs lags of two to three periods of the dependent variable as instruments for endogenous differences. Our regressions incorporate year and industry dummy variables, and standard errors are clustered at the firm level. To ensure the validity of the GMM estimator, we employ the Sargan test to control for over-identification issues and utilize the Arellano–Bond AR(2) test to examine the presence of second-order serial correlation in the first-differenced residuals.

Column (1) of Table 4 reports the results using SYS-GMM. The coefficient for departmental diversity is significantly positive at the 1% level, and the cross-product coefficients remain robust. With an AR(2) value of *p* of 0.122, exceeding the 0.05 threshold, we confirm the absence of second-order serial correlation in the first-differenced errors, thereby affirming the model’s specification. Similarly, a Sargan test value of *p* of 0.078, also exceeding the 0.05 threshold, confirms the validity of our instrumental variables.

**Table 4:** Endogenous control.

	(1)	(2)	(3)
Variables	*lnY_i,t + 1_*	*lnD_i,t_*	*lnY_i,t + 1_*
*lnH_i,t_*	−0.036		0.231***
	(0.033)		(0.074)
*lnD_i,t_*	0.941***		17.753***
	(0.308)		(4.228)
lnH_i,t_*lnD_i,t_	−0.054***		−0.950**
	(0.015)		(0.430)
*lnY_i,t_*	0.313**		
	(0.139)		
*University_num_i,t_*		0.006***	
		(0.002)	
Constant	−4.144	0.040**	3.450***
	(3.940)	(0.017)	(0.326)
Control	YES	YES	YES
Year	YES	YES	YES
Industry	YES	YES	YES
Observations	32,136	31,553	31,551
*R*-squared	–	0.328	0.144
AR(2) (*p*-value)	0.122		
Sargan test (*p-*value)	0.078		
Underidentification test (Kleibergen-Paap rk LM statistic)			37.568
			(0.000)
Weak identification test (*F* statistic)			19.653
10% maximal IV size			7.03
Endogeneity test			55.243
			(0.000)

Robust standard errors in parentheses ***p < 0.01, **p < 0.05, *p < 0.1.

To further mitigate endogenous concerns, we employ the instrumental variable method. The number of universities *(University_num)* in the city hosting the listed company is chosen as an instrumental variable due to two reasons. Firstly, a city’s number of universities is unlikely to directly affect local listed companies’ performance, fulfilling the exogeneity requirement. Secondly, listed Chinese firms, bearing certain social responsibilities, are encouraged to hire local university graduates. Concurrently, the synergistic collaboration between enterprises and local universities, often termed industry-academia-research cooperation, bolsters this trend. The presence of a larger number of local universities translates into a more diversified labor pool, thereby enabling listed companies to offer a broader spectrum of job roles. This, in turn, can potentially enhance the diversity within the corporate sector. This proposition aligns with the empirical findings of Guo et al. (2022), who posit that a diverse human capital structure, facilitated by a substantial number of local universities, can catalyze innovation within firms.

Table 4 column (2) presents the first stage regression results of the instrumental variable method, showing a significant positive correlation between the number of local universities and corporate sector diversity. Column (3) reports second stage regression results; the sign of *lnD_i,t_* remains positive, and the lnH_i,t_*lnD_i,t_ cross-term stays negatively significant, consistent with main regression results. The endogeneity test (value of *p*) shows endogeneity issues exist, but under-identification test (value of *p*) and weak instrumental variable test (*F* value) verify our chosen instrumental variables’ effectiveness. Therefore, even after attenuating potential endogenous influence, our empirical findings still back our proposed hypothesis.

To mitigate potential measurement errors that might arise from a few departments having many non-core employees, thereby skewing the diversity of departments, we have substituted the proxy variable for departmental diversity and conducted further empirical tests. The results are reported in Table 5. Here, *lnD1_i,t_* represents the logarithmic value of the ratio of the number of non-core departments a company has to the total number of departments, while *lnD2_i,t_* represents the logarithmic value of the ratio of the number of non-core departments a company has to the total number of non-core departments across all listed companies. These measures are used to gauge the intensity of a company’s departmental diversity in relation to itself and in comparison, to other listed companies, respectively.

**Table 5:** Robust test.

	(1)	(2)	(3)
Variables	*lnY_i,t + 1_*	*lnY_i,t + 1_*	*lnY_i,t + 1_*
*lnH_i,t_*	0.065***	0.055***	0.061***
	(0.009)	(0.008)	(0.008)
*lnD1_i,t_*	1.204***		
	(0.269)		
lnH_i,t_*lnD1_i,t_	−0.117***		
	(0.032)		
*lnD2_i,t_*		1.061***	
		(0.319)	
lnH_i,t_*lnD2_i,t_		−0.102***	
		(0.038)	
*lnD_i,t_*			1.121***
			(0.368)
lnH_i,t_*lnD_i,t_			−0.099**
			(0.044)
Constant	4.198***	4.219***	3.321***
	(0.186)	(0.187)	(0.149)
Control	Yes	Yes	Yes
Year	Yes	Yes	Yes
Industry	Yes	Yes	No
Observations	32,136	32,136	23,607
*R*-squared	0.742	0.741	0.757

Robust standard errors in parentheses ***p < 0.01, **p < 0.05, *p < 0.1.

As can be intuitively seen from *columns* (1) and (2), the coefficients of *lnD1_i,t_* and *lnD2_i,t_* are both significantly positive at the 1% level, and the interaction terms lnH_i,t_*lnD1_i,t_ and lnH_i,t_*lnD2_i,t_ are significantly negative. These results are consistent with those in Table 3, demonstrating that the main test results of this study remain robust even after replacing the explanatory variables.

Additionally, we observe that over 70% of the firms in our sample belong to the manufacturing sector. To further mitigate the influence of industry-specific characteristics and operational activities on the definition of ‘non-core departments,’ we narrow our research sample to observations solely from manufacturing sector as a robustness check. In this single-industry analysis, we forgo controlling for industry fixed effects. The results are reported in Column (3) of Table 5. The findings continue to demonstrate a significantly positive impact of departmental diversity on firm revenue, as well as a significant negative effect for the interaction term, thereby confirming the robustness of our results.

### 4.3 Further tests

To further elucidate the causal link between departmental diversity and firm performance, we consider a set of internal and external heterogeneity factors that might affect firm performance.

Firstly, a firm’s equity structure could modify departmental diversity’s impact on firm performance. Non-state-owned enterprises, with their operational and managerial agility, are potentially more adept at capitalizing on the knowledge spillover effects induced by departmental diversity. Moreover, non-state-owned enterprises typically prioritize efficiency and innovation, possibly encouraging inter-departmental knowledge exchange and collaboration, which could further enhance firm performance. This perspective finds support in Cui and Mak's (2002) research, which demonstrated superior performance in terms of innovation and efficiency among non-state-owned enterprises.

To test this, we introduce a dummy variable, *Soe_i,t._* If the firm is state-owned, *Soe_i,t_.* is set to 1; otherwise, it is set to 0. After regression with all explanatory variables, in Table 6, column (1) results show that, at a 1% significance level, non-state-owned enterprises exhibit greater firm performance enhancement due to departmental diversity.

**Table 6:** Further tests: the moderating effect of firm and market characteristics.

	(1)	(2)	(3)	(4)
Variables	*lnY_i,t + 1_*	*lnY_i,t + 1_*	*lnY_i,t + 1_*	*lnY_i,t + 1_*
*lnH_i,t_*	0.055***	0.042***	0.059***	0.055***
	(0.006)	(0.006)	(0.006)	(0.006)
*lnD_i,t_*	1.493***	0.887***	1.313***	1.391***
	(0.236)	(0.180)	(0.196)	(0.216)
lnH_i,t_*lnD_i,t_	−0.115***	−0.055**	−0.124***	−0.096***
	(0.031)	(0.026)	(0.027)	(0.031)
*Soe_i,t_*	0.209***			
	(0.048)			
lnH_i,t_*Soe_i,t_	0.007			
	(0.006)			
lnD_i,t_*Soe_i,t_	−0.630**			
	(0.273)			
lnH_i,t_*lnD_i,t_*Soe_i,t_	0.017			
	(0.040)			
*Market_i,t_*		0.437***		
		(0.062)		
lnH_i,t_* Market_i,t_		0.022***		
		(0.006)		
lnD_i,t_* Market_i,t_		0.568**		
		(0.230)		
lnH_i,t_*lnD_i,t_* Market_i,t_		−0.077***		
		(0.030)		
*Ma_age_i,t_*			0.178***	
			(0.040)	
lnH_i,t_* Ma_age_i,t_			−0.009	
			(0.005)	
lnD_i,t_* Ma_age_i,t_			−0.338	
			(0.237)	
lnH_i,t_*lnD_i,t_* Ma_age_i,t_			0.057*	
			(0.034)	
*Ma_degree_i,t_*				0.237***
				(0.040)
lnH_i,t_* Ma_degree_i,t_				−0.003
				(0.005)
lnD_i,t_* Ma_degree_i,t_				−0.530**
				(0.240)
lnH_i,t_*lnD_i,t_* Ma_degree_i,t_				0.006
				(0.035)
Constant	4.143***	4.184***	4.175***	4.152***
	(0.191)	(0.186)	(0.190)	(0.188)
Control	Yes	Yes	Yes	Yes
Year	Yes	Yes	Yes	Yes
Industry	Yes	Yes	Yes	Yes
Observations	32,136	32,136	32,136	32,136
*R*-squared	0.746	0.743	0.745	0.746

Robust standard errors in parentheses ***p < 0.01, **p < 0.05, *p < 0.1.

Secondly, the market environment might modify departmental diversity’s effect on firm performance. We introduce a dummy variable, *Market_i,t_,* to represent the economic environment’s upward or downward trend. This variable is determined by comparing the annual return rate of the CSI 300 Index with the average return rate of the Chinese A-share market. As column (2) illustrates, the positive impact of departmental diversity on firm performance is more pronounced during a booming stock market. Conversely, during a sluggish stock market, the combined effect of departmental diversity and corporate knowledge on reducing firm performance becomes more noticeable. This may be because, in economically prosperous periods, firms have more resources and opportunities to exploit the knowledge spillover effect facilitated by departmental diversity. However, during economic downturns, as per Bloom et al. (2018), firms might have to shift their focus towards cost control and efficiency enhancement, which could limit the impact of departmental diversity on firm performance.

According to the Upper Echelons Theory, executive traits can shape their strategic decisions (Hambrick and Mason, 1984). For instance, older executives may lean towards conservative strategies, potentially modulating the influence of departmental diversity on firm performance. To examine this, we define a binary variable, *Ma_age_i,t_.* It assumes a value of 1 if the firm’s average executive age exceeds the sample median; otherwise, it is 0. As results indicate, firms with older executive’s experience a less pronounced negative impact from departmental diversity via knowledge spillover. This may be attributed to the superior experience and managerial prowess often inherent in older executives, enabling them to effectively harness the firm’s knowledge assets and mitigate the adverse effects of departmental diversity on performance.

Furthermore, the education level of executives might sway the effects of departmental diversity on firm performance. Executive education is scored on a scale of 1 to 5, denoting, respectively, junior college and below, associate degree, bachelor’s degree, master’s degree (including MBA/EMBA), and doctoral degree. Subsequently, we compute the mean score of the executive team’s educational attainment and define the variable *Ma_degree_i,t_.* This assumes a value of 1 if the firm’s mean executive education level in year t surpasses the median for all firms in the same year; otherwise, it is 0. When cross-examined with the primary explanatory variable, the results suggest that departmental diversity’s performance-enhancing effect diminishes in firms where the average executive education level is above the median. This implies that higher executive education may offset the knowledge spillover attributable to departmental diversity. This could stem from the fact that more highly educated executives tend to possess more robust knowledge and skill sets, enabling them to effectively exploit and manage the firm’s knowledge assets, thereby dampening the impact of departmental diversity on firm performance. Wiersema and Bantel (1992) echoed these findings, identifying a significant correlation between executive education levels and corporate diversification strategies and performance.

In summary, factors including firm equity structure, market environment, and executive age and education levels may moderate departmental diversity’s impact on firm performance. These insights offer invaluable theoretical and practical implications for comprehending and leveraging departmental diversity to bolster firm performance.

## 5 Conclusion

This study analyzes the role of non-core department diversity in Chinese A-listed firms. Key findings include a positive correlation between department diversity and total income, suggesting that more diverse departments correspond with increased income. However, the intersection of departmental diversity and firm knowledge reveals diminishing returns, implying diversity benefits may be less noticeable in high R&D firms.

The study also highlights how different firm characteristics, like corporate ownership structure and executive demographics, moderate the impact of departmental diversity on performance. Despite its insights, the research’s focus on Chinese firms may limit the findings’ generalizability. Future research should consider diverse geographical and industrial contexts and explore the influence of department diversity on macroeconomic dynamics.

Overall, this research contributes to understanding non-core department diversity’s economic effects and paves the way for further investigation into department diversity, firm dynamics, and economic performance.

## Data availability statement

The original contributions presented in the study are included in the article/supplementary material, further inquiries can be directed to the corresponding author.

## Author contributions

SR: Conceptualization, Data curation, Formal analysis, Project administration, Resources, Writing – original draft. YW: Investigation, Methodology, Supervision, Validation, Visualization, Writing – review & editing.

## Footnotes

^1^This document was launched by the China Securities Regulatory Commission on February 17, 2023, and is a revised version of Guidance No. 1. It aims to regulate the information disclosure of initial public offerings and protect the legitimate rights and interests of investors.
